# Intracranial volumetric evaluation in postnatally repaired myelomeningocele infants

**DOI:** 10.1007/s00381-024-06444-2

**Published:** 2024-05-07

**Authors:** Hiroaki Hashimoto, Naoki Irizato, Osamu Takemoto, Yasuyoshi Chiba

**Affiliations:** 1 Department of Neurosurgery, Osaka Women’s and Children’s Hospital, Izumi, Osaka 594–1101 Japan; 2 Department of Neurological Diagnosis and Restoration, Graduate School of Medicine, Osaka University, Suita, 565–0871 Osaka Japan

**Keywords:** CT, Myelomeningocele, Postnatal repair, Volumetric measurement

## Abstract

**Introduction:**

Most myelomeningocele (MMC) cases present with ventriculomegaly or hydrocephalus, yet a comprehensive volumetric assessment of MMC intracranial structures is lacking. This study aimed to provide baseline data on intracranial structural volumes immediately after birth in MMC infants who underwent repair surgeries after birth (postnatal repair).

**Methods:**

In this retrospective single-center study, we analyzed 52 MMC infants undergoing postnatal repair, utilizing head computed tomography scans at birth for volumetric assessment. Intracranial volume (ICV), lateral ventricles volume (LVV), choroid plexus volume (CPV), and posterior cranial fossa volume (PCFV) were measured. Hydrocephalus was classified into no hydrocephalus, progressive hydrocephalus, and hydrocephalus at birth. Comparative analysis employed the Wilcoxon rank-sum test. Receiver operating characteristic (ROC) analysis discriminated cases with and without ventriculoperitoneal shunt (VPS).

**Results:**

The median values were 407.50 mL for ICV, 33.18 mL for LVV, 0.67 mL for CPV, and 21.35 mL for PCFV. Thirty-seven cases (71.15%) underwent VPS. ROC analysis revealed an LVV cut-off value of 6.74 mL for discriminating cases with and without VPS. Progressive hydrocephalus showed no significant difference in ICV but significantly larger LVV compared to no hydrocephalus. Hydrocephalus at birth demonstrated statistically larger ICV and LVV compared to the other two types.

**Conclusion:**

Baseline volumetric data were provided, and volumetric analysis exhibited statistical differences among three hydrocephalus types. These findings enhance our understanding of intracranial volumetric changes in MMC, facilitating more objective assessments of MMC cases.

**Supplementary Information:**

The online version contains supplementary material available at 10.1007/s00381-024-06444-2.

## Introduction

The most severe form of open spina bifida (dysraphism), myelomeningocele (MMC) [1], accounts for 98.8% of open spina bifida [2]. Hydrocephalus poses a significant concern [3], with comorbidity rates in MMC rising from 10–15% at birth to 65–85% within a few weeks [4–6]. A recent study has classified hydrocephalus treatment timing into simultaneous MMC repair with hydrocephalus treatment and delayed treatment [7]. These findings underscore the presence of progressive hydrocephalus in some MMC cases, which may remain undiagnosed at birth. As early as the 1970s, MMC cases were categorized into three hydrocephalus types: no hydrocephalus, progressive hydrocephalus, and hydrocephalus at birth [4]. However, quantitative volumetric assessment for these types is lacking, and concrete volumetric criteria for lateral ventricle volume to indicate shunt treatment in MMC infants are yet to be established.

Volumetric data on intracranial structures in MMC cases are lacking, and existing studies use ratios without establishing precise volume data [8–10]. This study aims to establish intracranial volumetric data calculated from head computed tomography (CT) scans of MMC cases who underwent repair procedures after birth (postnatal repair).

## Methods

### Patients and study setting

In this retrospective study, we included patients with MMC treated at our department from April 2006 to July 2023. All patients received head and spinal CT imaging after birth and underwent postnatal repair at our department. We specifically analyzed the initial head CT conducted immediately after birth. Before or after the repair surgeries, all cases underwent head and lumbar magnetic resonance imaging (MRI) to assess syringomyelia and Chiari malformation type II.

### Data collection

We analyzed various medical variables, including sex, gestational week, birth weight, birthplace, fetal diagnosis (FD), cesarean section (CS), days of CT imaging, and information related to MMC and ventriculoperitoneal shunt (VPS). MMC lesions were categorized into four types based on the involved vertebrae: thoracic type (involving at least one thoracic vertebra), upper lumbar type (focused among 1st and 3rd lumbar vertebrae), lower lumbar type (mainly among 3rd and 5th lumbar vertebrae), and sacral type (focally presented in sacral vertebra). A lumbosacral type, where the lesion ranges from the lower lumbar to sacral vertebrae (e.g., from the fifth lumbar to the third sacral vertebrae), was categorized into the lower lumbar type. The vertebrae count affected by MMC lesions was measured using spinal CT and MRI imaging. The MMC lesions were defined as neural placode or impairment of epithelialization.

Hydrocephalus comorbidity in MMC was categorized into three types: no hydrocephalus (no VPS required), progressive hydrocephalus (developing ventricles enlargement after birth, requiring VPS), and hydrocephalus at birth (obvious hydrocephalus at birth, treated by VPS). The decision on the necessity of VPS was determined by tense fontanelle or increasing head circumstance [11].

**Fig. 1:**
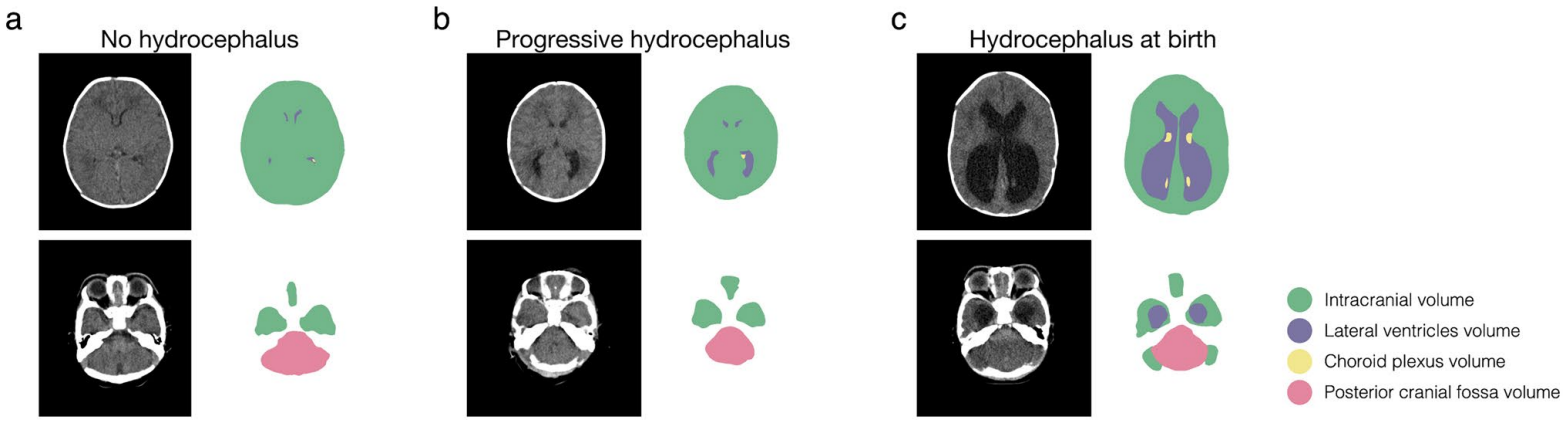
Representative segmentations. Segmentations for intracranial volume (ICV), lateral ventricles volume (LVV), choroid plexus volume (CPV), and posterior cranial fossa volume (PCFV) are colored as green, purple, yellow, and pink, respectively. Representative segmentations are presented from cases of no hydrocephalus (**a**), progressive hydrocephalus (**b**), and hydrocephalus at birth (**c**)

Quantitative assessment of intracranial volume (ICV), lateral ventricles volume (LVV), choroid plexus volume (CPV), and posterior cranial fossa volume (PCFV) in MMC patients used MATLAB R2023a (MathWorks, Natick, MA, USA) with digital imaging and communication in medicine (DICOM) CT data obtained immediately after birth. The target areas were manually segmented using the image segmenter app in MATLAB (https://www.mathworks.com/help/images/ref/imagesegmenter-app.html). Representative segmentation of ICV, LVV, CPV, and PCFV are shown in Fig. 1. Volume calculations (in milliliter, mL) were performed, and this method aligns with our previous studies [12–15]. LVV represented the volume of bilateral ventricles, and choroid plexus at the lateral ventricles and the foramen of Monro were assessed.

### Statistical analyses

Categorical data were presented as frequencies (percentages), while continuous variables were expressed as mean ± SD for normally distributed variables and as median with 1st-3rd quartiles for non-normally distributed variables. Spearman correlation coefficients were used to assess parameter correlations, and the chi-squared test compared two categorical variables. The unpaired T-test was employed for parametric distributions. The Kruskal-Wallis and Wilcoxon rank-sum tests were utilized for non-parametric distributions. Bonferroni correction was applied for multiple comparisons. P-values < 0.05 and corrected p-values < 0.05 were considered statistically significant. The receiver operating characteristic (ROC) curve determined the area under the curve (AUC) and cut-off values using the maximal Youden index (sensitivity + specificity – 1). Statistical analyses were performed using MATLAB R2023a’s Statistical and Machine Learning Toolbox.

## Results

### Baseline characteristics

Fifty-two MMC cases (27 females, 51.92%) were followed up for 8.11 (3.95 – 11.66) years as of September 30, 2023 (Supplemental Fig. 1). Baseline characteristics are presented in Table 1. Among MMC-lower lumbar type, 90% were lumbosacral type. The delivery method was statistically relevant to MMC lesion types; 91.67% of thoracic types were delivered by CS, compared to 40.00% of sacral types (chi-squared test, p < 0.001). Progressive hydrocephalus and hydrocephalus at birth occurred in 14 (26.92%) and 23 (44.23%) patients, respectively, leading to VPS surgeries in 71.15% of cases.

**Table 1:** Baseline data acquired from all enrolled infants with MMC

**Sex (n)**	
Male (%)	25 (48.08)
Female (%)	27 (51.92)
**Gestational week**^a^	38.10 ± 1.50
**Weight at birth (g)**	2870.46 ± 547.15
**Birthplace (n)**	
Our hospital (%)	38 (73.08)
Others (%)	14 (26.92)
**Fetal diagnosis (n)**	
Yes (%)	37 (71.15)
No (%)	15 (28.85)
**Cesarean section (CS)**^b^** (n)**	
Yes (%)	24 (50.00)
No (%)	24 (50.00)
**Reasons for CS**^c^** (n)**	
Breech presentation (%)	11 (45.83)
MMC (%)	5 (20.83)
Fetal head-pelvic imbalance (enlarged head circumference) (%)	3 (12.50)
Previous cesarean section (%)	2 (8.33)
Placental abruption (%)	1 (4.17)
Prolonged labor (%)	1 (4.17)
Marginal placenta previa (%)	1 (4.17)
Fetal bradycardia (%)	1 (4.17)
**Days of life at the initial CT**	0.00 (0.00 – 1.00)
**Days of life at MMC repair**	1.00 (0.50 – 2.00)
**Days from the CT to MMC repair**	1.00 (0.00 – 1.00)
**MMC lesion type (n)**	
Thoracic type (%)	12 (23.08)
Upper lumbar type (%)	3 (5.77)
Lower lumbar type (%)	10 (19.23)
Sacral type (%)	27 (51.92)
**MMC lesion vertebrae count**	3.50 (3.00 – 5.50)
**Syringomyelia (n)**	
Yes (%)	20 (38.46)
No (%)	32 (61.54)
**Chiari II malformation (n)**	
Yes (%)	30 (57.69)
No (%)	22 (42.31)
**Necessity of VPS (n)**	
Yes (%)	37 (71.15)
No (%)	15 (28.85)

*CT* computer tomography, *MMC* myelomeningocele, *VPS* ventriculoperitoneal shunt

^a^Missing data were observed in one case; the result was obtained from 51 cases

^b^Missing data were observed in four cases; the results were obtained from 48 cases

^c^Duplication was observed in one case; total counts resulted in 25 cases

### Correlation analysis between continuous variables

**Fig. 2:**
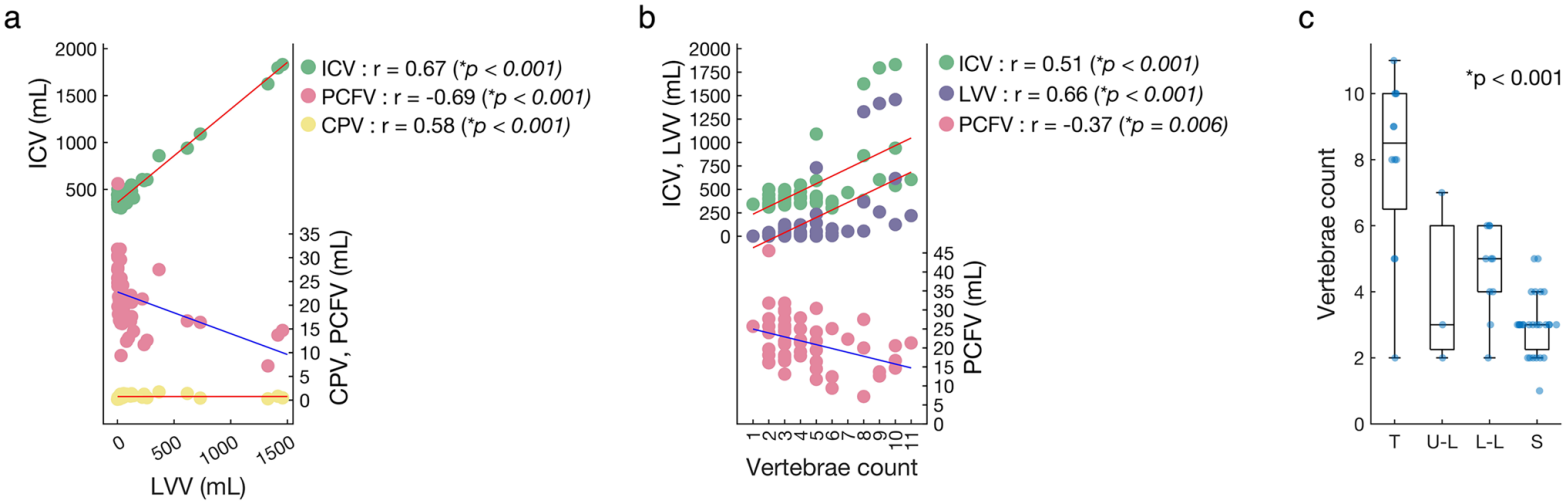
Continuous variable relationships. Scatter plots show correlations between lateral ventricles volume (LVV) and intracranial volume (ICV), posterior cranial fossa volume (PCFV), and choroid plexus volume (CPV) (**a**), and between vertebrae count and ICV, LVV, and PCFV (**b**). The correlation coefficients are denoted as “r”. Red lines indicate positive correlations, while blue lines indicate negative correlations. Myelomeningocele lesion vertebrae count distributions among lesion types are shown in a box-and-whisker plot overlaid with a bee-swarm plot (**c**). T, thoracic type; U-L, upper lumbar type; L-L, lower lumbar type; S, sacral type

Correlation analysis revealed positive correlations between gestational week and both weight at birth (r = 0.50, p < 0.001) and ICV (r = 0.32, p = 0.02). Weight at birth also positively correlated with ICV (r = 0.57, p < 0.001). In volumetric measurements, LVV demonstrated positive correlations with ICV (r = 0.67, p < 0.001) and CPV (r = 0.58, p < 0.001), and a negative correlation with PCFV (r = -0.69, p < 0.001) (Fig. 2a). MMC lesion vertebrae count exhibited positive correlations with ICV (r = 0.51, p < 0.001), LVV (r = 0.66, p < 0.001), and CPV (r = 0.29, p = 0.03), and a negative correlation with PCFV (r = -0.37, p = 0.006) (Fig. 2b). Significant differences in vertebrae count among MMC lesion types were noted (Kruskal-Wallis test, p < 0.001), with thoracic type having the highest counts compared to other types (Fig. 2c).

### Volumetric comparison

The median values were 407.50 mL for ICV, 33.18 mL for LVV, 0.67 mL for CPV, and 21.35 mL for PCFV (Table 2). MMC cases with VPS showed significantly larger volumes in ICV, LVV, and CPV, and a significantly lower volume in PCFV compared to those without VPS (using the Wilcoxon rank-sum test) (Table 2). Additionally, the association between VPS requirement and FD was evaluated. FD was statistically associated with VPS requirement; 83.78% of MMC cases with FD required VPS, compared to 40.00% of MMC cases without FD (chi-squared test, p = 0.002). MMC cases with FD showed significantly larger volumes in ICV, LVV, and CPV, and a significantly lower volume in PCFV compared to those without FD (using the Wilcoxon rank-sum test) (Table 2).

**Table 2:** Volumetric comparison among MMC cases with VPS vs. without VPS and with fetal diagnosis vs. without fetal diagnosis

**Parameters**	**MMC** (n = 52)	**VPS** (n = 37)	**Non-VPS** (n = 15)	***p-*value**	**with FD** (n = 37)	**without FD** (n = 15)	***p-*value**
ICV (mL)	407.50 (353.55 – 490.45)	421.20 (358.50 – 558.70)	373.90 (342.80 – 408.30)	*0.04	413.90 (358.50 – 558.70)	388.00 (339.70 – 417.38)	*0.04
LVV (mL)	33.18 (6.32 – 120.67)	55.21 (30.59 – 161.50)	3.53 (2.34 – 4.94)	* < 0.001	53.48 (24.23 – 161.50)	5.22 (3.33 – 22.86)	* < 0.001
CPV (mL)	0.67 (0.42 – 1.11)	0.96 (0.60 – 1.21)	0.34 (0.28 – 0.53)	* < 0.001	0.83 (0.51 – 1.20)	0.48 (0.31 – 0.59)	*0.01
PCFV (mL)	21.35 (16.65 – 25.60)	18.10 (15.75 – 21.80)	27.50 (24.80 – 30.32)	* < 0.001	19.70 (16.35 – 22.78)	25.00 (21.45 – 27.65)	*0.03

Median values are presented with 1st-3rd quartiles for each segmentation data. Using the Wilcoxon rank-sum test, statistical significance is determined at p < 0.05, denoted by an asterisk

*CPV* choroid plexus volume, *FD* fetal diagnosis, *ICV* intracranial volume, *LVV* lateral ventricles volume, *mL* milliliter, *MMC* myelomeningocele, *PCFV* posterior cranial fossa volume, *VPS* ventriculoperitoneal shunt

### Hydrocephalus type impact

MMC cases with VPS included those with progressive hydrocephalus and hydrocephalus at birth, while those not requiring VPS corresponded to the no hydrocephalus type. Statistical relevance was observed in hydrocephalus types and MMC lesion types (Supplemental Fig. 2a, chi-squared test, p = 0.002) and Chiari malformation comorbidity (Supplemental Fig. 2b, chi-squared test, p < 0.001). In hydrocephalus at birth type, thoracic lesion type and Chiari malformation comorbidity were predominant.

**Fig. 3:**
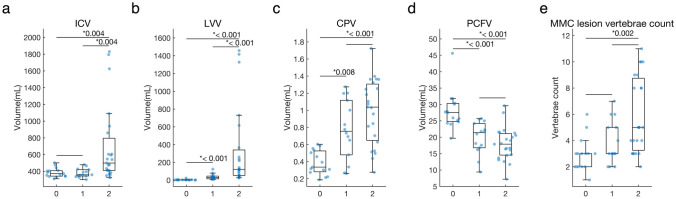
Differences among three hydrocephalus types. The box-and-whisker plot overlaid with beeswarm plots is presented for intracranial volume (ICV) (**a**), lateral ventricles volume (LVV) (**b**), choroid plexus volume (CPV) (**c**), posterior cranial fossa volume (PCFV) (**d**), and Myelomeningocele (MMC) lesion vertebrae count (**e**). The Wilcoxon rank-sum test is used, and acquired p-values are corrected by Bonferroni correction, multiplied by three, for the solution of multiple comparisons. Statistically significant corrected-p values < 0.05 are denoted with an asterisk. Hydrocephalus types are presented as 0 for no hydrocephalus, 1 for progressive hydrocephalus, and 2 for hydrocephalus at birth

While hydrocephalus at birth type showed significantly larger ICV than the others, there were no statistical differences between no hydrocephalus and progressive hydrocephalus (Fig. 3a). In LVV, even progressive hydrocephalus showed a significantly larger volume than no hydrocephalus (Fig. 3b). In CPV, both progressive hydrocephalus and hydrocephalus at birth demonstrated significantly larger volumes than no hydrocephalus; however, there was no statistical difference between these two types (Fig. 3c). Conversely, PCFV in no hydrocephalus exhibited significantly larger values than those in other two types (Fig. 3d). According to MMC lesion vertebrae count, hydrocephalus at birth demonstrated significantly larger values than no hydrocephalus (Fig. 3e). For exact values, refer to Supplemental Table 1.

### ROC analysis

**Fig. 4:**
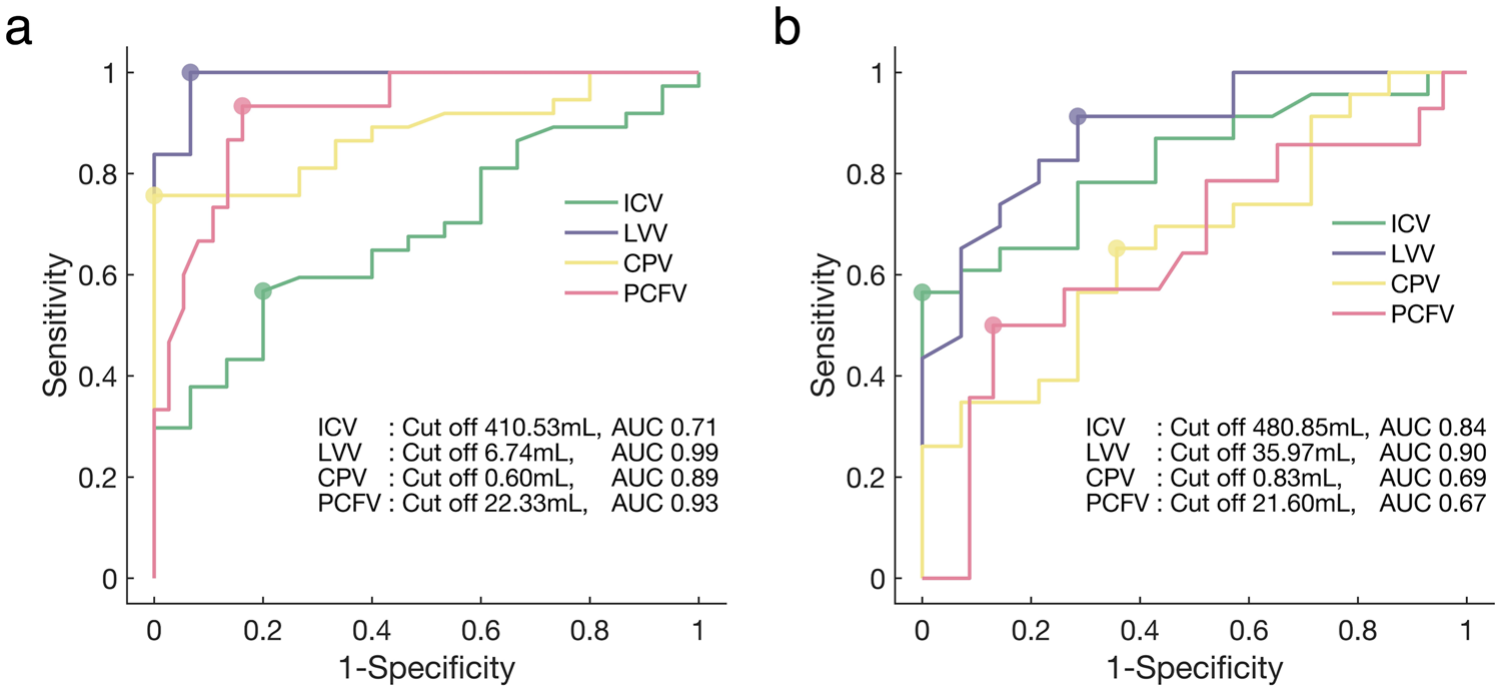
Receiver operating characteristic (ROC) curves. **a** ROC curves are calculated for discriminating cases requiring ventriculoperitoneal shunt (VPS) from those not requiring VPS using intracranial volume (ICV), lateral ventricles volume (LVV), choroid plexus volume (CPV), and posterior cranial fossa volume (PCFV). **b** ROC curves are calculated for discriminating cases with progressive hydrocephalus from those with hydrocephalus at birth using ICV, LVV, CPV, and PCFV

To distinguish cases with VPS from those without VPS, ROC analysis revealed the highest AUC value in LVV (AUC = 0.99), with a cut-off value of 6.74 mL (sensitivity 1.00, specificity 0.93) (Fig. 4a). Similarly, for discriminating cases with progressive hydrocephalus and hydrocephalus at birth, LVV also exhibited the highest AUC value (AUC = 0.90), with a cut-off value of 35.97 mL (sensitivity 0.91, specificity 0.71) (Fig. 4b).

### DICOM slice width

The width of DICOM slices varied from 1.00 mm to 6.98 mm, with a median value of 5.00 (5.00 – 5.33) mm. Slice width correlated positively with both ICV (r = 0.38, p = 0.006) and LVV (r = 0.40, p = 0.003) (Supplemental Fig. 3a and b). However, there was statistical significance between hydrocephalus types and slice width (chi-squared test, p = 0.02) (Supplemental Fig. 3c). Among patients with hydrocephalus at birth, who showed a statistically larger volume of ICV and LVV than the other two types, slice widths of 1 or 3 mm were not utilized at all. Thus, there was unevenness in the utilized slice width among hydrocephalus types. We mentioned this issue related to width-variation in the Discussion.

## Discussion

Established baselines for intracranial structural volumes in MMC infants are lacking. In this study, we provide volumetric measurement values of ICV, LVV, CPV, and PCFV calculated from postnatally repaired MMC infants. Additionally, we demonstrated statistical differences in volumetric values among three hydrocephalus types: no hydrocephalus, progressive hydrocephalus, and hydrocephalus at birth. Moreover, the LVV cut-off value distinguishing between MMC cases with and without VPS was 6.74 mL.

The reported VPS rate for MMC with postnatal closure ranges from 60–80% [7, 11, 16–23], consistent with our results at 71.15%. We observed that 28.85% had no hydrocephalus, 26.92% had progressive hydrocephalus, and 44.23% had hydrocephalus at birth, with these three hydrocephalus types having been identified as early as the 1970s [4]. Progressive hydrocephalus following postnatal closure is believed to result from inadequate CSF resorption after repair surgery [17]. While the quantitative differences among these three types have remained unclear, we successfully demonstrated them using volumetric measurements. It is predictable that hydrocephalus at birth showed significantly larger ICV and LVV than the other two, and no hydrocephalus demonstrated significantly larger PCFV than the other two. However, it is interesting that progressive hydrocephalus showed no statistical differences in ICV compared to no hydrocephalus but significantly larger LVV than no hydrocephalus. This means that even in CT images taken immediately after birth, the characteristics of progressive hydrocephalus, where ICV is the same but LVV is higher than in no hydrocephalus, are apparent.

In a previous study on postnatal MMC closure, symptoms linked to hydrocephalus, such as apneic and bradycardic episodes, did not show statistical relevance to the need for CSF diversion. However, fontanelle characteristics, head circumference at birth, and the rate of head growth were significantly associated with CSF diversion [11]. Despite the absence of established volume criteria for VPS indication, our ROC analyses revealed a cut-off value of 6.74 mL for LVV in the necessity of VPS. This value, derived from our single-center experience, currently lacks scientific validation as a VPS criterion. Nevertheless, we anticipate that it could serve as a valuable reference for physicians managing MMC with hydrocephalus.

Our results on the correlations between MMC lesion vertebrae count and measured volumes reveal a clear trend: the more severe the MMC lesion (indicated by a higher vertebrae count), the smaller PCFV and the larger LVV. The thoracic type had the largest vertebrae count, signifying greater severity than the other three types. These findings align with previous studies, where larger spinal defects correlated with increased ventricular size [24]. The level of the lesion significantly affected the incidence of shunting, with more cephalad lesions correlating with higher rates: 97% in the thoracic, 87% in the lumbar, and 68% in the sacral spine [25]. In our study, all MMC cases with the thoracic type required treatment with VPS, while the rate of VPS in the sacral type was 51.85% (14/27 cases).

Small posterior fossa was observed in 84% of infants with MMC [19]. In a fetus with MMC, CSF leakage into the sac or amniotic fluid is thought to lead to hindbrain herniation, known as Chiari II malformation [26]. Hindbrain herniation is believed to play a key role in inducing hydrocephalus through increased resistance to cerebral venous outflow or abnormalities in CSF absorption or flow [5, 22, 27, 28]. Therefore, it is reasonable to infer that MMC induces small PCFV, leading to the enlargement of LVV and subsequent hydrocephalus. Thus, the negative correlation between PCFV and LVV in our study is acceptable and aligns with previous findings on the negative correlation between posterior cranial fossa hypoplasia and ventriculomegaly [10]. Our results also demonstrated a positive correlation between ICV and LVV, and we infer that the enlargement of LVV caused the enlargement of ICV.

Additionally, a positive correlation between LVV and CPV was observed, consistent with prior studies on this relationship [14]. Considering that the choroid plexus is a soft tissue, we infer that small ventricles compress the choroid plexus, while large ventricles induce a larger volume of the choroid plexus by releasing compressive pressure. Therefore, there is a possibility of underestimation or overestimation of CPV influenced by the size of the ventricles. In various neurological diseases such as Alzheimer’s disease [29] and stroke [30], some papers have reported the importance of the choroid plexus in neuropathology. However, it remains unknown how MMC pathology affects the growth of choroid plexus volume. Further research is needed to elucidate whether the larger size of CPV observed in larger LVV is a neuropathological result or merely an overestimation.

Our correlation analysis revealed positive correlations between ICV and slice width and between LVV and slice width, supporting concerns about overestimation. However, a statistical unevenness emerged between hydrocephalus types and the slice width; all slice widths for hydrocephalus at birth were over 5 mm, with none at 1 mm to 3 mm. This means that slice widths of 1 mm to 3 mm were only used in cases of no hydrocephalus or progressive hydrocephalus. Considering that ICV and LVV of hydrocephalus at birth were significantly larger than those of no hydrocephalus or progressive hydrocephalus, it is reasonable to conclude that the statistical distributional unevenness among hydrocephalus types and slice widths affected the positive correlation among them. Additionally, as there were no correlations between slice width and PCFV, or CPV, we judged that the influence of the non-consistency of slice width was minimal. However, it is crucial to note that this non-consistency of the utilized CT scans’ slice width is a significant limitation of this study.

## Limitations

Firstly, conducting a single-center and retrospective posed challenges in ensuring consistency in slice width. Secondly, we lacked concrete criteria for VPS operation, and VPS procedures were performed based on empirical judgment by neurosurgeons in our department. Further studies are needed to establish a reliable and clinically feasible LVV criterion for VPS. Finally, there may be overestimation or underestimation when calculating CPV.

## Conclusions

We presented intracranial volumetric data obtained immediately after birth from infants with MMC who underwent postnatal repair and highlighted volumetric differences among three hydrocephalus types. Our data could serve as a valuable reference for a more objective assessment of hydrocephalus in MMC cases.

### Supplementary Information

Below is the link to the electronic supplementary material.

Supplementary file1 (PDF 2927 KB)

## Data Availability

No datasets were generated or analysed during the current study.

## Abbreviations

AUC: Area under the curve
CPV: Choroid plexus volume
CS: Cesarean section
CSF: Cerebrospinal fluid
CT: Computed tomography
DICOM: Digital imaging and communication in medicine
FD: Fetal diagnosis
ICV: Intracranial volume
LVV: Lateral ventricles volume
mL: Milliliter
MMC: Myelomeningocele
MRI: Magnetic resonance imaging
PCFV: Posterior cranial fossa volume
ROC: Receiver operating characteristic
SD: Standard deviation
VPS: Ventriculoperitoneal shunt
