# Supplementary material for: Intracranial volumetric evaluation in postnatally repaired myelomeningocele infants
Source: Childs Nerv Syst. 2024 May 7;40(9):2851–8. doi: 10.1007/s00381-024-06444-2 (PMC11322201; doi:10.1007/s00381-024-06444-2)
Supplement: Supplementary file 1 — Supplementary file1 (PDF 2927 KB) [file 381_2024_6444_MOESM1_ESM.pdf]

Title: Intracranial volumetric evaluation in postnatally repaired myelomeningocele infants

Authors: Hiroaki Hashimoto, MD, Ph.D[1, 2*], Naoki Irizato, MD[1], Osamu Takemoto, MD, Ph.D[1], Yasuyoshi Chiba, MD, Ph.D[1]

Affiliations:

1. Department of Neurosurgery, Osaka Women's and Children's Hospital, Izumi, Osaka 594–1101, Japan
2. Department of Neurological Diagnosis and Restoration, Graduate School of Medicine, Osaka University, Suita, Osaka 565–0871, Japan

Corresponding Author's name and current institution: Hiroaki Hashimoto
Department of Neurosurgery, Osaka Women's and Children's Hospital, Izumi, Osaka 594–1101, Japan.
Corresponding Author's Email: h-hashimoto@ndr.med.osaka-u.ac.jp

Supplemental Fig.1 Patients enrollment in this study.

The flow chart outlines the inclusion criteria for patients with MMC. A total of 52 patients are analyzed, although data are missing for a gestational week and cesarean section in some cases. Among these, 37 patients require VPS due to hydrocephalus, including 14 with progressive hydrocephalus and 23 with hydrocephalus at birth.

MMC, myelomeningocele; VPS, ventriculoperitoneal shunt.

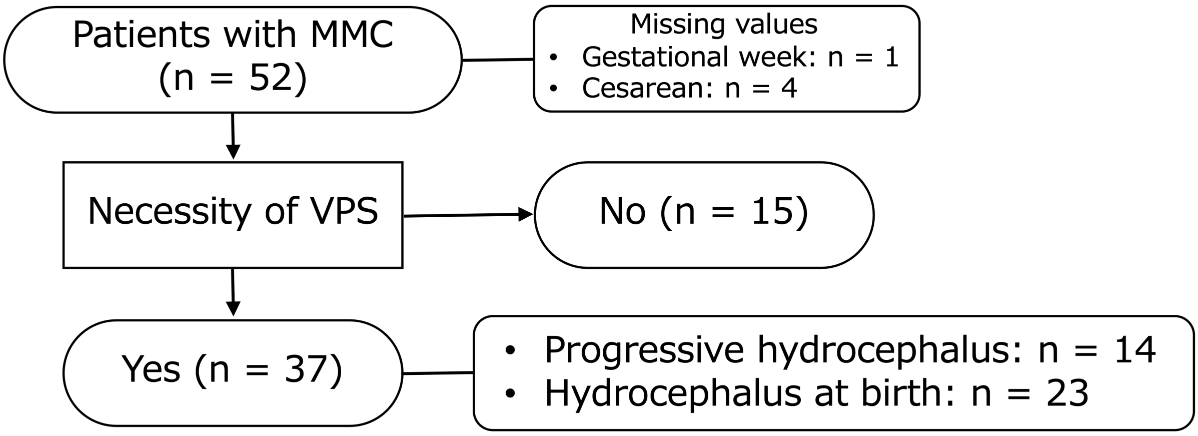

Supplemental Fig.2 Hydrocephalus types heatmap charts.

The distribution of patients among hydrocephalus types and MMC lesion types is presented in a heatmap chart in panel (a) and among hydrocephalus types and comorbidity with Chiari malformation (Yes = 1, No = 0) in panel (b). Hydrocephalus types are denoted as 0 for no hydrocephalus, 1 for progressive hydrocephalus, and 2 for hydrocephalus at birth. MMC lesion types are indicated as T for thoracic type, U-L for upper lumbar type, L-L for lower lumbar type, and S for sacral type.

MMC, myelomeningocele.

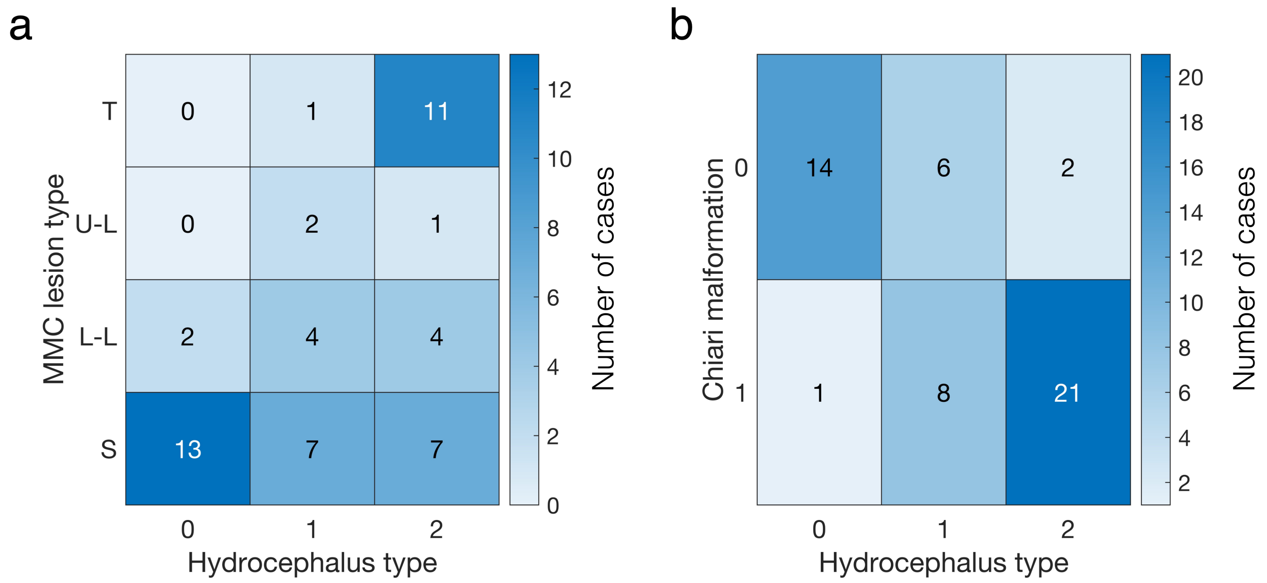

Supplemental Fig.3 DICOM slice width utilized in this study.

Scatter plots depicting the relationships between slice width and ICV and LVV are presented in panels (a) and (b). Depending on hydrocephalus types, the scatter plots are color-coded in green, blue, and red. The correlation coefficients are denoted as "r". Red lines indicate positive correlations. Panel (c) displays the distribution of patients among hydrocephalus types and slice width groups in a heatmap chart. Hydrocephalus types are marked as 0 for no hydrocephalus, 1 for progressive hydrocephalus, and 2 for hydrocephalus at birth. The utilized slice width is divided into two groups, including 1 to 3mm or over 5mm.

DICOM, digital imaging and communication in medicine; ICV, intracranial volume; LVV, lateral ventricles volume.

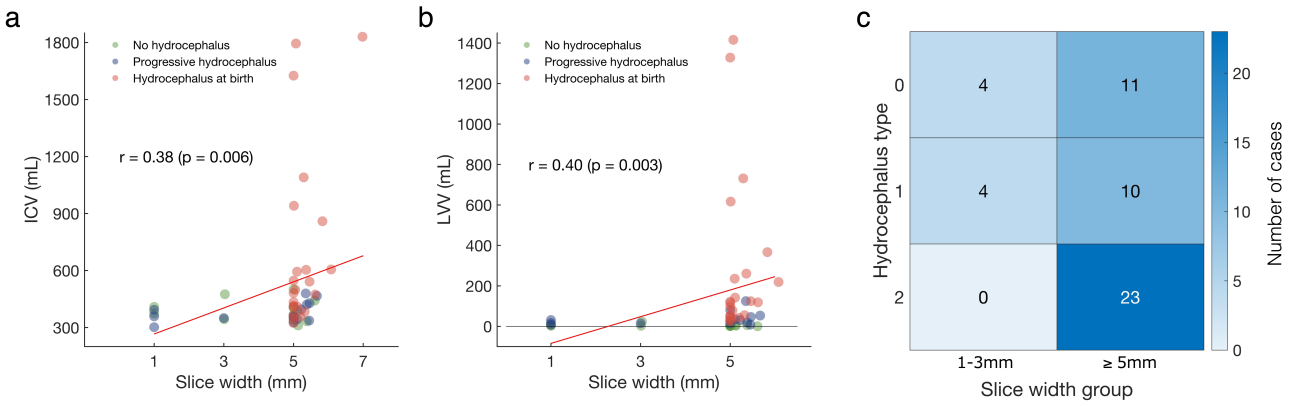

Supplemental Table 1 Median values with 1st-3rd quartiles presented in Figure 3.

	**No hydrocephalus**	**Progressive hydrocephalus**	**Hydrocephalus at birth**
**ICV (mL)**	373.90 (342.80 – 408.30)	359.60 (344.30 – 421.20)	499.20 (410.02 – 796.18)
**LVV (mL)**	3.53 (2.34 – 4.94)	30.31 (15.25 – 46.19)	122.51 (53.91 – 340.22)
**CPV (mL)**	0.34 (0.28 – 0.53)	0.76 (0.48 – 1.12)	1.04 (0.65 – 1.31)
**PCFV (mL)**	27.50 (24.80 – 30.32)	21.40 (16.80 – 24.20)	17.90 (14.55 – 21.12)
**MMC lesion vertebrae count**	3.00 (2.00 – 3.00)	3.00 (3.00 – 5.00)	5.00 (3.25 – 8.75)

CPV, choroid plexus volume; ICV, intracranial volume; LVV, lateral ventricles volume; mL, milliliter; MMC, myelomeningocele; PCFV, posterior cranial fossa volume.
